# Cardiovascular magnetic resonance detects microvascular dysfunction in a mouse model of hypertrophic cardiomyopathy

**DOI:** 10.1186/s12968-021-00754-z

**Published:** 2021-05-31

**Authors:** Min-Chi Ku, Frank Kober, Yi-Ching Lai, Andreas Pohlmann, Fatimunnisa Qadri, Michael Bader, Lucie Carrier, Thoralf Niendorf

**Affiliations:** 1 Berlin Ultrahigh Field Facility (B.U.F.F.), Max Delbrück Center for Molecular Medicine in the Helmholtz Association (MDC), Robert-Rössle Strasse 10, 13125 Berlin, Germany; 2 DZHK (German Centre for Cardiovascular Research), Partner site Berlin, Berlin, Germany; 3 Centre de Résonance Magnétique Biologique et Médicale (CRMBM), Aix-Marseille University, CNRS, Marseille, France; 4 Molecular Biology of Peptide Hormones, Max Delbrück Center for Molecular Medicine in the Helmholtz Association (MDC), Berlin, Germany; 5 Department of Experimental Pharmacology and Toxicology, University Medical Center Hamburg-Eppendorf, Hamburg, Germany; 6 DZHK (German Centre for Cardiovascular Research), Partner site Hamburg/Kiel/Lübeck, Berlin, Germany; 7 Experimental and Clinical Research Center (ECRC), A Joint Cooperation between the Charité Medical Faculty and the Max-Delbrück Center for Molecular Medicine, Berlin, Germany

**Keywords:** Hypertrophic cardiomyopathy (HCM), Myocardial remodelling, Microvascular dysfunction, Perfusion, Myocardial blood flow (MBF), Cardiac MRI (CMR), Arterial spin labelling (ASL)

## Abstract

**Background:**

Hypertrophic cardiomyopathy (HCM) related myocardial vascular remodelling may lead to the reduction of myocardial blood supply and a subsequent progressive loss of cardiac function. This process has been difficult to observe and thus their connection remains unclear. Here we used non-invasive myocardial blood flow sensitive CMR to show an impairment of resting myocardial perfusion in a mouse model of naturally occurring HCM.

**Methods:**

We used a mouse model (DBA/2 J; D2 mouse strain) that spontaneously carries variants in the two most susceptible HCM genes—*Mybpc3 and Myh7* and bears the key features of human HCM. The C57BL/6 J (B6) was used as a reference strain. Mice with either B6 or D2 backgrounds (male: n = 4, female: n = 4) underwent cine-CMR for functional assessment at 9.4 T. Left ventricular (LV) wall thickness was measured in end diastolic phase by cine-CMR. Quantitative myocardial perfusion maps (male: n = 5, female: n = 5 in each group) were acquired from arterial spin labelling (cine ASL-CMR) at rest. Myocardial perfusion values were measured by delineating different regions of interest based on the LV segmentation model in the mid ventricle of the LV myocardium. Directly after the CMR, the mouse hearts were removed for histological assessments to confirm the incidence of myocardial interstitial fibrosis (n = 8 in each group) and small vessel remodelling such as vessel density (n = 6 in each group) and perivascular fibrosis (n = 8 in each group).

**Results:**

LV hypertrophy was more pronounced in D2 than in B6 mice (male: D2 LV wall thickness = 1.3 ± 0.1 mm vs B6 LV wall thickness = 1.0 ± 0.0 mm, *p* < 0.001; female: D2 LV wall thickness = 1.0 ± 0.1 mm vs B6 LV wall thickness = 0.8 ± 0.1 mm, *p* < 0.01). The resting global myocardial perfusion (myocardial blood flow; MBF) was lower in D2 than in B6 mice (end-diastole: D2 MBF_global_ = 7.5 ± 0.6 vs B6 MBF_global_ = 9.3 ± 1.6 ml/g/min, *p* < 0.05; end-systole: D2 MBF_global_ = 6.6 ± 0.8 vs B6 MBF_global_ = 8.2 ± 2.6 ml/g/min, *p* < 0.01). This myocardial microvascular dysfunction was observed and associated with a reduction in regional MBF, mainly in the interventricular septal and inferior areas of the myocardium. Immunofluorescence revealed a lower number of vessel densities in D2 than in B6 (D2 capillary = 31.0 ± 3.8% vs B6 capillary = 40.7 ± 4.6%, *p* < 0.05). Myocardial collagen volume fraction (CVF) was significantly higher in D2 LV versus B6 LV mice (D2 CVF = 3.7 ± 1.4% vs B6 CVF = 1.7 ± 0.7%, *p* < 0.01). Furthermore, a higher ratio of perivascular fibrosis (PFR) was found in D2 than in B6 mice (D2 PFR = 2.3 ± 1.0%, B6 PFR = 0.8 ± 0.4%, *p* < 0.01).

**Conclusions:**

Our work describes an imaging marker using cine ASL-CMR with a potential to monitor vascular and myocardial remodelling in HCM.

## Background

Hypertrophic cardiomyopathy (HCM) is the most prevalent cardiac genetic disease with an estimated population prevalence of HCM ranging from 1:200 to 1:500 individuals [1, 2] and mainly caused by distinct genetic variants in sarcomeric proteins. The major clinical feature of HCM is left ventricular (LV) hypertrophy (LVH), which is often asymmetric and preferentially affects the interventricular septum, in the absence of any other conditions that could induce LVH, such as environmental factors (stress or physical activities), hypertension, or aortic stenosis [9]. Sudden cardiac death can be the first manifestation of HCM, particularly in young [3, 4] and asymptomatic patients [5, 6]. The key sarcomere genes [7] most frequently affected genes in HCM are the cardiac myosin-binding protein C (*MYBPC3*) [8] and β-myosin-heavy chain (*MYH7*) [9]. Aberrant proteins trigger myocardial tissue remodelling which is a complex process of transcriptional, signaling, structural, electrophysiological, and functional events occurring within the cardiac myocyte and myocardium [10]. The myocardial tissue remodelling contributes to small vessel disease (namely vascular remodelling), cardiac hypertrophy, myocyte disarray, myocardial fibrosis and ultimately compromise the cardiac function [11, 12]. While the early detection of myocardial remodelling is a key to effective disease management [13], at present no in vivo imaging markers of dynamic changes of microvasculature have been found [14]. CMR can detect ventricular wall hypertrophy and fibrosis progression, two main features that are useful in predicting HCM prognoses [15], but monitoring those morphological changes on the macroscopic and mesoscopic scales cannot detect subclinical microvascular dysfunctions, which potentially might be more sensitive markers of disease progression [16]. Here we focused on the remodelling of small vessels and microvascular dysfunction in the myocardium, which may lead to a reduction of myocardial blood supply and a subsequent progressive loss or deterioration of cardiac function. In response to varying physiological or pathological conditions and functional demands, blood vessels are continuously adapting their structural change [12].

Microvascular dysfunction in HCM patients has been proposed as a strong predictor of clinical outcomes and mortality risk [17], but the relationship between this and myocardial remodelling is poorly understood. The small vessels of the microvascular network supply the myocardium with oxygen to maintain functional cardiac tissue integrity [18]. Myocardial wall thickening connected to pressure overload is thought to play a role in impairing coronary inflow and trigger a progressive ventricular dysfunction [19]. Hypertrophy-induced deficits in myocardial perfusion and micro-circulation might also trigger the pathological deposition of collagen in the myocardium. This reduces myocardial blood flow and might be the key determinant of subsequent heart failure [20]. This suggests that monitoring changes in micro-perfusion might make an effective early indicator to diagnose the disease and have the chance to provide early procedures that improve blood flow.

Local myocardial blood volume and blood flow are important indicators of changes in micro-perfusion [21]. Recent work has investigated the relationship between hypertrophy and the myocardial microvascular network in HCM [22]. In these studies, the fractional tissue blood volume per cardiac tissue volume (Bvf), defined as the volume in the microvascular network, was directly probed by Bvf-sensitive CMR using blood oxygenation-weighted imaging contrast [22]. Myocardial regions with reduced Bvf were highly correlated with ventricular wall thickening [22, 23]. Yet the factors responsible for the reduction of microvascular network volume remain unknown.

We hypothesize that vascular dysregulation caused by hypertrophy or excessive collagen deposits may contribute to the microstructural changes in HCM and cause the reduction. Using a mouse model that closely reflects human HCM, we quantified the amount of blood delivered to myocardial tissue per unit time using myocardial blood flow (MBF) CMR based on the labelling of arterial proton spins [24, 25]. Unlike widely used methods based on contrast agent enhanced first pass perfusion CMR, arterial spin labelling (ASL)-CMR does not require an injection of exogenous contrast material, and thus can be repeated [26]. Employing this approach, the goal was to find any sign of small vessel change indicated by myocardial perfusion and then correlate the changes detectable by CMR with microstructural histology in the murine heart – something that is difficult to do in human patients. For this purpose MBF, capillary density, perivascular fibrosis and interstitial fibrosis were determined.

## Methods

### Mouse model carries HCM gene variants

**Fig. 1:**
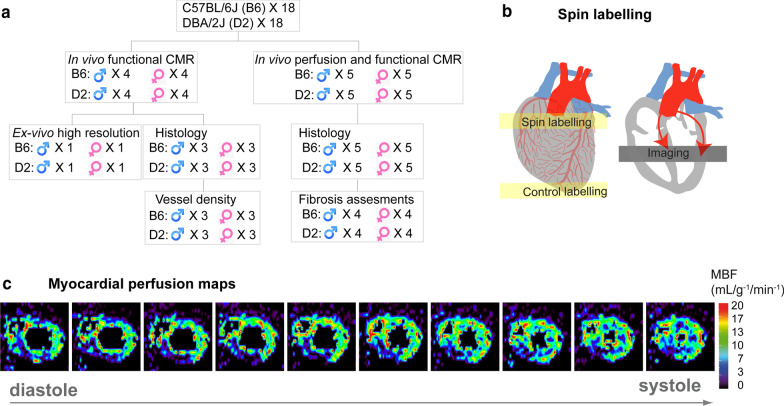
**a** Schematic representation of experimental design. **b** Schematic illustration of the tagging slice position used for quantifying myocardial blood flow (MBF) with arterial spin labelling (ASL)-cardiovascular magnetic resonance (CMR). By labelling water molecules in the inflowing blood from coronary artery as tracers which then perfused into myocardium, labelled blood signal can be imaged in the mid-left ventricle (LV). **c** Ten consecutive color coded short axis myocardial perfusion maps from a measurement series over one cardiac cycle

All animal studies were approved by the Berlin State review board at the ‘’Landesamt für Gesundheit und Soziales (LAGeSo; State Office for Health and Social Affairs Berlin)’’. Genetic variants that potentially cause myocardial functional changes were previously reported [27]. We used a mouse model (DBA/2J; D2 mouse strain) that spontaneously carries variants in the two most susceptible HCM genes—*Mybpc3 and Myh7* and bears the key features of human HCM [28]. The C57BL/6J (B6) strain was used as reference. Mice were handled according to the LAGeSo and internal (MDC) rules and regulations. In total, 36 eight-month-old mice were used (4 male and 4 female mice with B6 or D2 backgrounds were used for cardiac functional assessment and 5 male and 5 female mice with B6 or D2 were used for ASL-CMR). The experimental design is illustrated in Fig. 1a.

### Cardiovascular magnetic resonance imaging

#### Physiological control

All in vivo CMR measurements were performed on a 9.4 T small animal CMR system (Biospec 94/20, Bruker Biospin, Germany). Anesthesia of mice was induced using 3% isoflurane (CP-Pharma, Germany) in 500 mL/min medical air and 500 mL/min oxygen and was maintained at 0.5 to 1.0% isoflurane after the induction. During CMR examinations, it was important to maintain hemodynamic stability to avoid confounding interference with the imaging results. During cine ASL-CMR scans, oxygen was given at a dose of at least 800 mL/min to avoid hypoxia. Core body temperature was maintained at 36 ± 0.5 °C using a heated water tubing system. Heart rates, respiration rates and core body temperature were closely monitored using a remote monitoring system (Model 1025, SA Instruments Inc., Stony Brook, New York, USA).

#### Cardiac physiological assessments

For the high-fidelity approaches for CMR in mice, image acquisition was conducted using a cryogenically-cooled radiofrequency (RF) transceiver antenna (CryoProbe, Bruker Biospin). To obtain a stack of cardiac short axis (SAX) views covering the whole mouse heart, 9–10 slices were consecutively acquired using a self-gated bright-blood cine (IntraGate-FLASH, TR/TE = 8.5/1.58 ms, FA = 20°, receiver bandwidth = 98 kHz, FOV = 11 × 22 mm^2^, matrix size = 192 × 384, slice thickness = 0.8 mm, with a temporal resolution of 16 images per cardiac cycle [29]. Cardiac function assessment was performed using cvi42 (Circle Cardiovascular Imaging, Calgary, Alberta, Canada) and analyzed on a slice-by-slice basis. Endo- and epi-cardiac borders were manually segmented in end-systole and end-diastole using a stack of SAX cine images. LV ejection fraction (LVEF) and myocardial mass (both in diastole and systole) were calculated. LV wall thickness was measured in end diastolic phase. Six SAX views were used for measuring the maximal wall thickness.

#### Quantification of myocardial perfusion using cine ASL-CMR (cineASL)

The cineASL technique used is described in detail elsewhere [24, 30]. This cineASL approach was previously validated against a classical FAIR Look-Locker Gradient-Echo technique under rest and stress conditions [30] and built upon a previously published version. The principle of ASL-CMR is to label the inflowing coronary arterial blood as an endogenous diffusible tracer (Fig. 1b). It relies on an electrocardiographically (ECG)-gated cine Fast Low Angle Shot (cine-FLASH) technique repeated over several cardiac cycles for each line of k-space. In a preparation module, spin labelling using dedicated radiofrequency pulses is applied to tag (by inverting the spins) the longitudinal magnetization of coronary blood water protons before it enters the imaging plane in the myocardium [25]. The labelling pulses were produced by replacing one cine readout within the cardiac cycle at the time of valve closure (end systole) by a hyperbolic secant adiabatic inversion pulse. For labelling, a slice-selective inversion slab was placed on the aortic root while another selective inversion slab was placed in the opposite direction below the imaging slice (as control labelling). Two cardiac gated (on ECG) mid-LV SAX cine image series were acquired under labeled and control conditions. For averaging, the acquisition was repeated 35 times over several cardiac cycles for labeled and control conditions without a delay, as described in the original version [30]. Provided that the magnetization of the inflowing blood is the only difference between the control and the label image, a difference map was calculated yielding perfusion-weighted images of the heart for each cardiac cine phase, with the signal intensity being proportional to myocardial perfusion. For quantification of MBF, the acquisitions were done using a 72 mm inner diameter volume resonator (Bruker Biospin) for uniform RF transmission in conjunction with a 4 channel cardiac RF array (Bruker Biospin) used for signal reception. The following parameters were used for the cineASL technique [24]: TR/TE = 7.6/1.12 ms, FA = 6°, FOV = 25 × 25mm^2^, matrix size = 128 × 64, slice thickness = 1 mm, temporal resolution within one cardiac cycle = 7.6 ms, 35 averaged cine blocks for both tag and control images. Throughout the ASL measurements, a two lead electrode was used to detect ECG signals of the mouse heart. The ECG trace was monitored with dedicated modules for small animals (SA Instruments Inc.). Image acquisition was gated upon detection of the ECG’s R-wave. Quantitative image analysis and calculation of perfusion maps were performed using in-house developed analysis tools built in the Interactive Data Language (IDL). After averaging the steady-state cine repetitions from successive cardiac cycles, regional perfusion was analyzed using a ventricular segmentation model [31]. The border of each myocardial segment was manually delineated for mid-ventricular SAx slices obtained from end-diastolic and end-systolic cardiac phases. Mean MBF were then calculated for regions of interest defined by septal, lateral, anterior, and inferior traced endocardial and epicardial contours which are illustrated in Fig. 4a.

#### *High resolution CMR of *ex vivo* heart*

Following in vivo CMR, mice were perfused with 4% PFA. Fixed ex vivo hearts (n = 2 from each group for illustration) were imaged with high spatial resolution to check for the presence of myocardial hypertrophy and disarray (RARE, TR/TE = 2200/40.7 ms, FOV = 15 × 10 mm^2^, matrix size = 500 × 336, slice thickness = 0.3 mm, in-plane spatial resolution = 30 µm).

#### Histological assessment and microscopic image processing

Directly after the ASL-CMR scans, the mouse hearts were quickly removed from the chest and perfused with ice-cold cardioplegic solution (15 mM KCl in PBS). This step arrested the cardiac cycle during diastole. Heart tissue were post fixed in 4% PFA solution and then dehydrated. Fixed hearts were either cryopreserved in a 30% sucrose-PBS solution for at least 3 days at 4 °C or embedded with paraffin. Cryopreserved hearts were then embedded in OCT (Tissue-Tek, Sakura Finetek Germany GmbH, Staufen im Breisgau, Germany), frozen on dry ice, and stored in − 80 °C until sectioning. For tissue sectioning, 10 µm cross-sections of hearts were cryo-sectioned using a cryostat microtome (Leica CM3050S, Wetzlar, Germany) starting from the apex. Four µm paraffin sections of heart were prepared using a microtome (Thermo Fischer HM355S, Fisher Scientific GmbH, Schwerte, Germany).

#### Fluorescent microscopy

For detecting myocardial microvasculature, heart sections were analyzed using immunofluorescence (IF) staining methods. Briefly, 10 µm cryo-preserved sections were mounted on glass slides. For blocking the unspecific bindings, tissue sections were treated with a blocking solution containing 10% normal donkey serum (NDS; Sigma-Aldrich, Taufkirchen, Germany), 4% fetal bovine serum (BSA; Sigma-Aldrich, Germany), 0.2% Triton X-100 in PBS for 3 h at room temperature. Heart tissue sections from entire mid-ventricular short-axis sections were then incubated with Alexa Fluor 488 conjugated Isolectin B4 (1:500; Invitrogen) diluted in 0.01 M PBS with 5% NDS over night at 4 °C. After washing in freshly prepared PBS with 2.5% NDS, the nuclei were stained with Hoechst 33,342 (Sigma-Aldrich). The presence of fluorescent stained cells was determined by observation on a microscope (BZ 9000 Keyence GmbH, Neu-Isenburg, Germany). Quantifications of Isolectin B4 + vessels were performed in 10 random fields captured under 200 magnifications. All image processing was then performed using ImageJ (National Institutes of Health, Bethesda, Maryland, USA) and Adobe Illustrator (Adobe, San Jose, California, USA). The captured images were carefully screened. Blurred images were excluded from the quantification. For quantification, small vessel density in myocardium was analyzed using ImageJ software plugins (vessel density) in five random microscopic fields in the whole myocardium and expressed as percentage (the number of small vessels per square millimeter tissue).

#### Light microscopy

To determine the interstitial collagen (collagen volume fraction; CVF) [32] and perivascular collagen deposit (perivascular fibrosis ratio; PFR) [33] in the mouse heart, cryopreserved or rehydrated heart paraffin sections were stained in Sirius Red solution (detecting collagen) for 1 h at room temperature. Stained sections were rinsed twice in 1% acetic acid, dehydrated through serial concentrations of ethanol washes, cleared in xylene, and mounted with a xylene based mounting medium. Extensive washes were performed between each step. Images were taken using a microscope (BZ-9000, Keyence Corporation of America, Itasca, Illinois, USA). CVF was determined as the percent of collagen-stained area/total myocardial area and quantified using ImageJ software. Excluding endocardial and perivascular collagen, the interstitial CVF (%) was calculated from the area stained by the Sirius red. PFR (%) was determined from all collagen surrounding an intra-myocardial coronary artery and was calculated as the ratio of the fibrosis area surrounding the vessel to the total vessel area. For each sample, 5 microscopic fields were examined.

### Statistical analysis

All data analyses of the cardiac physiological measurements using CMR and histopathology were performed by reviewers blinded to the mouse genetic makeup. Experimental analysis and statistics were conducted in R studio (R Studio Inc., Boston, USA; http://www.rstudio.com/) or GraphPad Prism 5 (GraphPad Software, La Jolla, California, USA). The graphs were generated by GraphPad Prism 5. The comparison between B6 and D2 mice in diastolic and systolic cardiac phases were analyzed using one-way ANOVA. The sampling frequency was further tested for normality using Kolmogorov–Smirnov test. A low K-S statistic value represent with a high p-value indicate that data is normally distributed. Statistical difference of MBF by sex or cardiac phases between groups were further tested using factorial analysis of variance (Factorial ANOVA) followed by post-hoc Turkey HSD (Honestly Significant Difference). The Spearman's rank correlation coefficient was used for testing the strength of the correlation between LV thickness and global MBF. Capillary density (%), CVF (%) and PFR (%) from B6 and D2 were compared using *t*-test followed by Mann Whitney test. All data are presented as mean ± SD. The differences were considered statistically significant at *P < 0.05; **P < 0.01; and ***P < 0.001.

## Results

### Cardiac functional measurements in spontaneous HCM mouse model

**Fig. 2:**
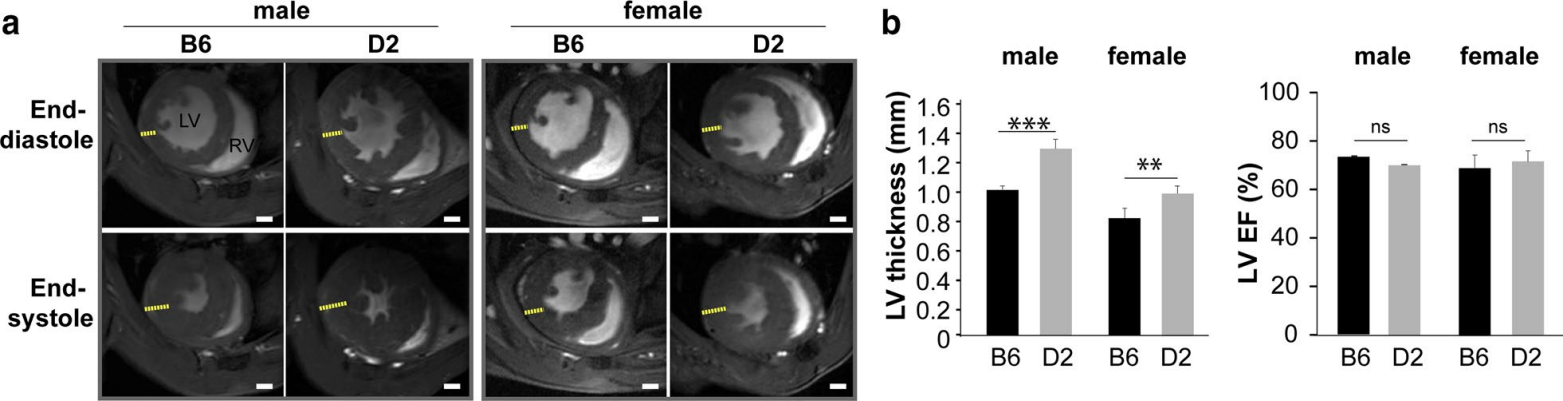
Hypertrophic cardiomyopathy (HCM) mice carrying *mybpc3* and *myh7* gene variants show myocardial hypertrophy. **a** Representative FLASH cine images (upper row: end-diastole; lower row: end-systole) show mid-ventricular short axis (SAX) views in male B6 (C57BL/6) and D2 (DBA/2J) mouse heart acquired at 9.4 T. Yellow lines outline the thickness of left ventricle (LV). White scale bar = 1 mm. **b** cine-CMR revealed preserved LV function in D2 HCM mice (male: n = 4, female: n = 4) compared to B6 (male n = 4, female n = 4). Functional assessments (LV thickness, LV ejection fraction (LVEF), data are shown as mean ± SD) were done on a slice-by-slice basis. Endo- and epi-cardiac borders were manually segmented in end-systole and end-diastole using a stack of short axis FLASH cine images. LVEF were calculated for both sexes. Data are shown as the mean ± SD. **p < 0.01, ***p < 0.001 using one-way ANOVA with Tukey’s multiple-comparisons test

LVH is a key phenotype of HCM. We evaluated the cardiac phenotype in mice carrying natural genetic variants in *Mybpc3* and *Myh7,* which are the two most frequently mutated genes in human HCM [34]. In all mice groups, LV remodelling was not observed in B6 control animals. D2 mice exhibited LVH when compared to B6 reference strain (Fig. 2a). LVH was more pronounced in both male and female D2 mice than in B6 mice (male: D2 LV wall thickness = 1.3 ± 0.1 mm vs B6 LV wall thickness = 1.0 ± 0.03 mm, *p* < 0.001; female: D2 LV wall thickness = 1.0 ± 0.1 mm vs B6 LV wall thickness = 0.8 ± 0.1 mm, *p* < 0.01) (Fig. 2b). LVEF in D2 mice remained normal and showed no significant difference when compared to B6 (male: D2 LVEF = 70.3 ± 5.8% vs B6 LVEF = 73.8 ± 0.7%; female: D2 LVEF = 71.9 ± 4.1%, vs B6 LVEF = 68.9 ± 5.5%; Fig. 2b).

### Cine ASL-CMR detection of myocardial perfusion deficit

**Fig. 3:**
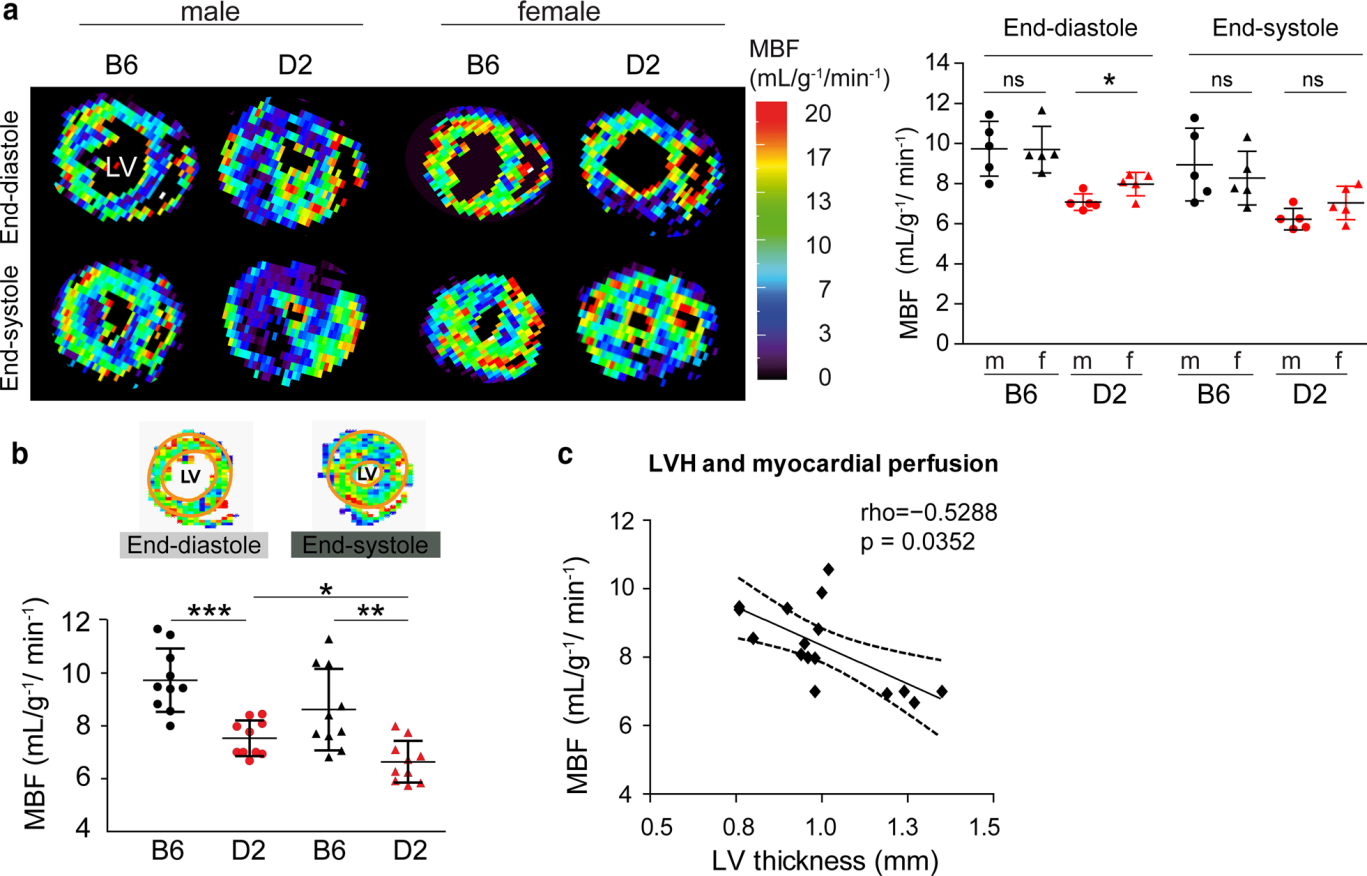
Myocardial perfusion deficits in HCM mice. **a** Representative MBF maps showing end-diastolic (upper row) and end-systolic (lower row) cardiac phases for a mid-ventricular SAX views in both male and female control B6 and HCM (D2) mouse heart. MBF maps were acquired using cineALS CMR which facilitates the monitoring of dynamic MBF changes in the cardiac cycles. No sex difference in dynamic MBF changes in the cardiac cycles was observed between groups (B6 male n = 5, B6 female n = 5, D2 male n = 5, D2 female n = 5). Data are shown as the mean ± SD. **b** Mean MBF were calculated for region of interest (ROI, depicted in orange colour by manually traced endocardial and epicardial contours) in mid-ventricular end-diastole and end-systole in all control (B6) and HCM (D2) mice (n = 10 for each group). Data are shown as the mean ± SD. **p < 0.01, ***p < 0.001 using one-way ANOVA with Tukey’s multiple-comparisons test. (c) Negative Spearman’s rank correlations were seen between left ventricular thickness and myocardial blood flow (n = 16)

We investigated non-invasively the impact of primary genetic variants on myocardial perfusion using MBF sensitive CMR. As previously shown, the cine ASL-CMR sequence allows tracking of dynamic MBF changes across the entire cardiac cycle [24], which is necessary to depict any cyclic change in microvascular function in the myocardium. For this purpose we divided the cardiac cycle into 14–17 cardiac phases (depending on the heart rates of individual mice) and allowed depicting the end-diastole and end-systole separately (Fig. 3a, left). No significant differences between males and females were found. In D2 females, a higher end diastolic MBF was noted compared to D2 males (P < 0.05), but no other significant differences were observed (Fig. 3a, right). The resting global myocardial perfusion in the mid-ventricular slice was lower in D2 than in B6 mice (end-diastole: D2 MBF_global_ = 7.5 ± 0.6 vs B6 MBF_global_ = 9.3 ± 1.6 ml/g/min, *p* < 0.05; end-systole: D2 MBF_global_ = 6.6 ± 0.8 vs B6 MBF_global_ = 8.2 ± 2.6 ml/g/min, *p* < 0.01; Fig. 3b). Blood flow to the myocardium occurs mainly during diastole. We therefore compared the cyclic changes in blood flow in each group. In B6 control mice, the global myocardial perfusion did not differ between the end-systolic and end-diastolic phases (*p* = 0.090), whereas in D2 mice it was significantly lower in the end-systolic than in the end-diastolic phases (*p* < 0.05; Fig. 3b).

Moreover, there were correlations between the global MBF and overall LV wall thickness. A significant negative correlation was observed between the MBF at rest and the correspondent LV thickness, i.e., thicker myocardium was associated with lower myocardial perfusion (rho =  − 0.5288; *p* = 0.0352, non-parametric Spearman correlation; Fig. 3c).

### Resting myocardial perfusion deficit shows regional pattern

**Fig. 4:**
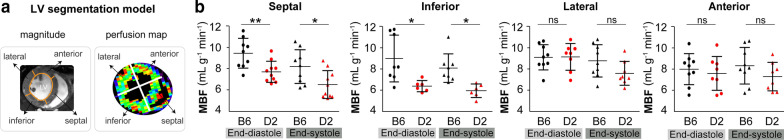
Quantitative myocardial perfusion at rest. **a** Schematic illustration of the LV segmentation model for a mid-ventricular SAX slice of the mice heart. **b** Mean MBF were calculated for interventricular septum for a mid-ventricular slice during end-diastole and end-systole in all control (B6; n = 9) and HCM (D2; n = 10) mice. Each dot represents an individual mouse. Data are shown as the mean ± SD. *p < 0.05, **p < 0.01, one-way ANOVA with Tukey’s multiple-comparisons test

Based on the segmentation model (Fig. 4a), we analysed the regional change of MBF. Septal myocardial perfusion was significantly lower in D2 than in B6 mice (end-diastole: D2 MBF_septal_ = 7.9 ± 1.0 vs B6 MBF_septal_ = 8.9 ± 1.7 ml/g/min, *P* < 0.01; end-systole: D2 MBF_septal_ = 8.8 ± 1.4 vs B6 MBF_septal_ = 6.9 ± 1.5 ml/g/min, *p* < 0.05; Fig. 4b). The inferior myocardial region adjacent to the septum showed lower MBF in D2 than in B6 mice (end-diastole: D2 MBF_inferior_ = 9.1 ± 2.2 vs B6 MBF_inferior_ = 6.4 ± 0.5 ml/g/min, *p* < 0.05; end-systole: D2 MBF_inferior_ = 8.1 ± 1.4 vs B6 MBF_inferior_ = 5.9 ± 0.6 ml/g/min, *p* < 0.05). Lateral and anterior regions did not show significant MBF difference.

### Detection of impaired microvasculature and fibrosis in HCM mice

**Fig. 5:**
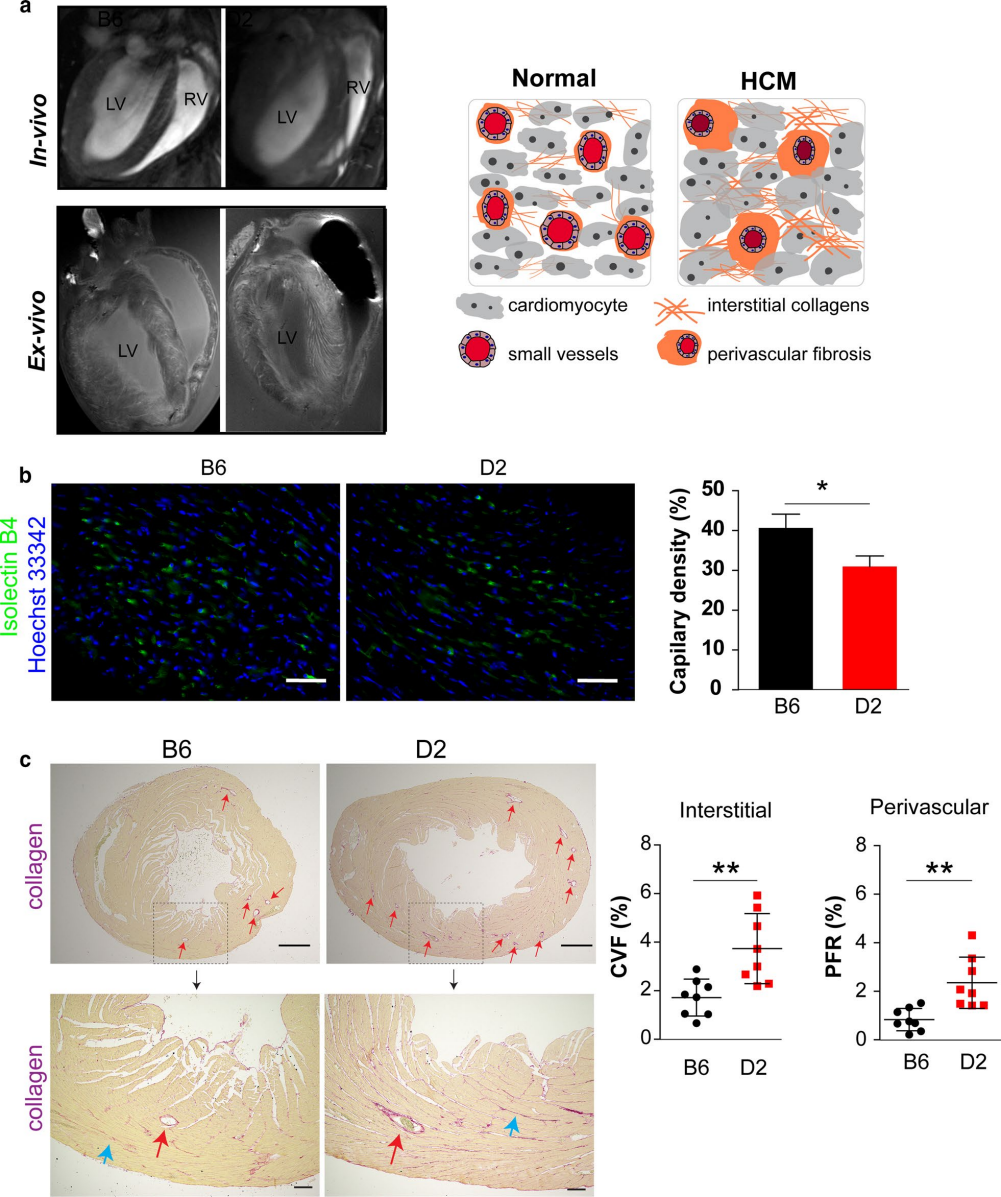
**a** Representative long axis view of in vivo (in-plane spatial resolution = 57 µm) and ex vivo (in-plane spatial resolution = 30 µm) high resolution images showing detailed myocardial structure indicating hypertrophy. Right: A schematic to illustrate the relationship of hypertrophy, interstitial fibrosis, perivascular fibrosis and microvasculature. **b** Representative immunofluorescence staining of mouse heart LV sections for Isolectin B4 and the quantification of the capillary density ratio (number/5 imaging area). Scale bar = 50 μm; Data are shown as the mean ± SD (n = 3 each sex groups; 6 B6 and 6 D2 mice, respectively). *P < 0.05 (*t*-test) **c** Sirius red staining show interstitial and perivascular collagen deposition. Upper row images scale bar = 500 µm; lower row images scale bar = 100 µm. Red arrow depicts perivascular fibrosis and blue arrow depicts interstitial fibrosis. Collagen volume fraction (CVF) and perivascular fibrosis ratio (PFR) in septal and inferior area of B6 (n = 8) and D2 (n = 8) mice left ventricle. Data are shown as the mean ± SD. **p < 0.01, using *t*-test with Mann Whitney test

The factors that might be involved in the reduction of small vessel function such as hypertrophy was determined (Figs. 2 and 3). We further confirmed the thickened LV and myofiber disarray by high resolution in vivo and ex vivo CMR in D2 mice (Fig. 5a). It is known that small vessels control local blood flow and impact the exchange functions of downstream segments of the myocardial microcirculation [21]. We therefore determined whether the small vessel density in the septal and inferior myocardium differ in those mice at steady state. Immunofluorescence staining for endothelial cells (with endothelial cell–specific Isolectin B4) revealed a lower number of Isolectin B4^+^ vessels in D2 than in B6 mouse myocardium (D2 capillary = 31.0 ± 3.8% vs B6 capillary = 40.7 ± 4.6%, *p* < 0.05; Fig. 5b).

To visualize the myocardial fibrosis precisely, we performed collagen staining to indicate the exact position of fibrosis within the LV (Fig. 5c). Myocardial interstitial collagen was characterized by the increase in the percentage of total LV myocardial tissue occupied by collagen staining. CVF was significantly higher in D2 than B6 LV (D2 CVF = 3.7 ± 1.4% vs B6 CVF = 1.7 ± 0.7%, *p* < 0.01). Furthermore, we also observed higher ratio of perivascular fibrosis in D2 mice versus B6 mice (D2 PFR = 2.3 ± 1.0%, B6 PFR = 0.8 ± 0.4%, *p* < 0.01). Microscopic characteristics revealed the relation of perfusion deficit with both perivascular and interstitial fibrosis.

## Discussion

In this study, we demonstrate that cine ASL-CMR is capable of detecting changes in myocardial perfusion that are correlated to a reduction in small vessel density in the myocardium. The reduced myocardial perfusion provides an indication for microvascular dysfunction in HCM. The myocardial perfusion deficiency is correlated with LVH and associated with myocardial fibrosis and loss of small vessel densities, features which are presumably mediated by the genetic variants commonly found in HCM. We analysed the global as well as the regional differences of MBF based on the cardiac segmentation model. However, motion artefacts have been recognized as a limiting factor for the precise detection of myocardial regional differences. These artefacts can be induced by myocardial contraction/relaxation, by respiratory motion causing a shift a in the chest wall and the diaphragm, by susceptibility changes due to motion of the heart–lung or heart–liver interface or by non-uniform regionally motion patterns (different twist and strain) of the heart [35]. Another confounding factor could be the different rotational behaviour during early systole in mice [31]. The assessment of regional differences in myocardial perfusion pattern has to be further validated with respect to their functional component.

Subclinical myocardial remodelling dictates the progression of HCM, but currently diagnosis and treatment of the disease are restricted to measurements of myocardial hypertrophy. Diagnosing microvascular dysfunction has been a challenge. Standard tests used to diagnose coronary artery disease are not designed to detect small vessel disease, so a better understanding of the factors associated with microvascular dysfunction and its relation to the development of HCM would represent a major step toward earlier diagnosis and better management of the disease. Our results can partially explain previous findings showing that HCM patients have lower myocardial blood volume than healthy counterparts [22]. In the presence of LVH, effective perfusion is critical to compensate for hypertrophy-induced micro-vessel restriction in the HCM heart. This hypothesis will need to be refined, tested and extended to obtain a fuller picture of the development of the pathological features of the disease and their consequences. In addition, ASL-CMR is not a standard clinical technique, while quantitative first-pass contrast agent based techniques are clinically established for myocardial perfusion imaging. Therefore, despite the necessity of bolus injections, robust quantitative first-pass post-processing methods are now becoming more widely available and currently represent the method of choice when similar studies are conducted in humans. *En route* to non-contrast myocardial perfusion imaging such as ASL-CMR, more studies are needed to prove that both methods are equally relevant for clinical applications [36].

Although a number of post-mortem studies have demonstrated marked impairment of the coronary microcirculation in the absence of significant coronary lesions in HCM patients [37, 38], the influence of genetic variants on microvascular function in HCM myocardium has remained unexplored. Measuring myocardial perfusion using CMR or other imaging modalities may reveal deficiencies in microvascular function even in mild cases or asymptomatic HCM patients, giving it potential as an independent predictor of clinical outcome [17, 39]. For instance, many patients with HCM have symptoms of myocardial ischemia and cardiac dysfunction. Abnormal intramural coronary arteries with markedly thickened walls and narrowed lumens are observed in HCM patients and may represent a genetic component of the underlying myocardial remodelling process [40]. Although the clinical relevance of microvascular dysfunction in HCM remains unclear, the fact that intramural coronary arteries exhibit structural alterations in areas of substantial myocardial fibrosis suggests a causal role for these arteries in producing ischemia [41]. In line with previous findings, we show in this study that the abnormal microvasculature was substantially pronounced in the inferior septum. Although it has become evident that perivascular fibrosis, but not interstitial fibrosis is associated with the impairment of coronary blood flow [42], we observed both perivascular and interstitial fibrosis are more pronounced in our HCM mouse model. Fibrosis is a dynamic process, thus the characterization of temporal pattern of both perivascular fibrosis and interstitial fibrosis requires additional attention [43].

Classically, HCM is characterized by varying degrees of LVH with a preserved or sometimes even increased LVEF [44]. Our data suggest that the quantification of cyclic changes of myocardial perfusion under resting conditions is sensitive to detect differences in HCM when using cineASL-based perfusion CMR. The incidence of heart failure (HF) in HCM patients is about ∼50%, with symptoms varying from mild to severe [45, 46]. Due to a substantial heterogeneity, ascertaining the incidence of which HF in HCM is challenging. In HCM, HF has two distinct clinical features: HFpEF or HFrEF. In the majority of HCM patients, HF is manifested as HFpEF phenotype, known as “diastolic heart failure” while only a minority develops HFrEF at a later stage. A recent large cohort study confirmed that systolic dysfunction (manifest by low LVEF) is highly associated with prognosis [47]. On the other hand, in HFpEF, microvascular dysfunction was evident as the major determinant of the pathological cascade that justifies clinical manifestations [48, 49]. Therefore it is extremely relevant to identify microvascular dysfunction, including the cause and its mechanisms. Recent reports have suggested that patients with HFpEF exhibit an increased incidence of small vessel disease as shown by abnormal blood flow and increased microvascular resistance [49, 50]. Our results support the idea that myocardial perfusion changes are connected to hypertrophy and the extent of fibrosis and thus can be an additional remodelling marker of the HCM phenotype regardless of cardiac dysfunction.

Blood flow through small vessels in the myocardium is influenced by changes in myocardial tissue pressure during heartbeats. Most myocardial perfusion occurs during the diastolic heart phase, when the myocardial pressure is low [51]. It may also be affected by total capillary density. Vascular endothelial dysfunction obviously impairs myocardial perfusion [52, 53]; our study shows that capillary density is a potential key confounding factor for myocardial perfusion.

The accuracy of this non-invasive MBF measurement using cineASL has already been validated against the more common FAIR Look-Locker Gradient Echo technique, which was in turn validated against fluorescent microspheres [54]. The variations of blood flow throughout the cardiac cycle under different conditions were previously assessed using cineASL [30]. Notably, cyclic variation of MBF across the cardiac cycle has been thoroughly assessed using this technique in rats [24]. Since the MBF value in mice has been shown to be strongly dependent on the anesthetic conditions, the relatively high values found here could be due to differences in anesthesia. The anesthesia is also affected by the individual experimental conditions: actual isoflurane concentration inhaled, which is dependent on the face mask, space around the animal and gas recovery and therefore difficult to match across different lab setups. Other influencing factors are temperature and breathe rate. Therefore, one has to be cautious that the results on MBF may not reflect perfect resting conditions due to the vasodilating effect of isoflurane.

Sex differences are known in several facets of cardiac physiology [55]. Sex differences in myocardial perfusion have previously been demonstrated in healthy subjects and in patients with nonischemic heart failure using positron emission tomography (PET) or CMR [56, 57]. These reports suggested that females have higher resting myocardial perfusion compared to males. Our data showed the tendency of higher resting MBF in both diastolic and systolic cardiac phases. The sex difference of myocardial perfusion will therefore need to be takin into account in future studies.

## Limitations

While we observed the sex differences in end-diastolic MBF in D2 mice but not in other experimental groups, this observation might be attributed to the small sample size used, which is a recognized limitation of our study. We are therefore unable to comment on the exact sex influence that might exist with perfusion abnormalities. To address this scientific question future work will need to incorporate a larger sample size. The cardiac function and MBF measurements were not derived from the same group of mice. Notably, the physiological effects of anaesthesia during CMR have to be minimized. Consequently, we have designed the CMR measurement protocol such that the total duration of an individual in vivo experiment does not exceed a maximum of 60 min. Therefore all measurements were performed in the same time window.

We noted that MBF values in mice found in this study are higher when compared with published findings [30, 58, 59]. Notwithstanding our findings remain comparable and within the error ranges given in the literature [60]. Multiple reasons can contribute to modifications of the resting perfusion in rodents such as the level of anesthesia, gas mixture or temperature. The comparatively high control values have also been obtained by Abdesselam et al. [58], who attributed this to a moderate cardiac stress condition due to isoflurane-dependent vasodilation to the elevated baseline values. In previous studies in healthy mice using ASL-CMR reported myocardial perfusion ranging from 5.0 ± 0.8 to 6.9 ± 1.7 ml/g/min [30, 59, 61] at 4.7 T and 7.0 ± 0.5 ml/g/min at 7 T [60]. Our experiments were performed at 9.4 T and revealed an average MBF of 8.2 ± 2.6 ml/g/min, which is in accordance with the literature values. No specific validation against gold-standard techniques has been done for perfusion measured in this study. However, the employed techniques were earlier validated in rats but not mice.

Our study did not tackle the influence of single genetic mutations. Therefore, the specific effects of *Mybpc3* or *Myh7* point mutations need to be pursued, as does the relation between oxygen consumption and myocardial perfusion in HCM.

To tackle the diastolic dysfunction, we did not include myocardial strain measurements in our study because we would like to first emphasize the early microstructural change such as vascular deficiency in HCM. MR tagging can be useful for detecting changes in myocardial strain in the mouse heart. We anticipate including this approach into our future studies. Our preliminary results provide the basis for future investigations of the entire disease course, including the early disease phase when the pathological changes are subtle. Then by performing a comprehensive protocol of CMR measurements including parametric mapping (T_1_, T_2_ and T_2_*) and ASL, we can observe how CMR biomarkers change over time, and how these changes can predict later disease outcomes.

## Conclusions

We used state-of-the-art in vivo CMR to evaluate microstructure and functional changes in mice with HCM genetic variants. We have demonstrated that small vessel dysfunction is present in these HCM variants in association with myocardial fibrosis. Our work thus suggests that more research on MBF as an imaging marker with a potential to monitor early myocardial microvascular remodelling is relevant in the field.

## Data Availability

The data derived from ASL-CMR are public and available under https://figshare.com/articles/figure/IDL_images/14251241. Our group is also initiator of Open Source MRI (http://www.opensourceimaging.org/). R script for statistical analysis of 4 myocardial regions is published on the GitHub (https://doi.org/10.5281/zenodo.4061597). Experimental design and data collection were handled according to the ARRIVE guidelines (https://www.nc3rs.org.uk/sites/default/files/documents/Guidelines/NC3Rs%20ARRIVE%20Guidelines%20Checklist%20%28fillable%29.pdf).

## Abbreviations

ASL: Arterial spin labelling
Bvf: Fractional tissue blood volume per cardiac tissue volume
CMR: Cardiovascular magnetic resonance
Cine ASL-CMR: Dynamic arterial spin labelling based CMR
CVF: Collagen volume fraction
ECG: Electrocardiogram
FLASH: Fast low angle shot
FOV: Field-of-view
HCM: Hypertrophic cardiomyopathy
HFpEF: Heart failure with preserved ejection fraction
HFrEF: Heart failure with reduced ejection fraction
LV: Left ventricle/left ventricular
LVEF: Left ventricular ejection fraction
LVH: Left ventricular hypertrophy
MBF: Myocardial blood flow
MYBPC3: Cardiac myosin-binding protein C
MYH7: β-Myosin-heavy chain
PFR: Perivascular fibrosis ratio
SAx: Short axis
TR: Repetition time
TE: Echo time
SD: Standard deviation
